# Evaluation of Dry Eye After Refractive Surgery According to Preoperative Meibomian Gland Status

**DOI:** 10.3389/fmed.2022.833984

**Published:** 2022-04-25

**Authors:** Qianwen Gong, Anqi Li, Lin Chen, Huijuan Chen, Jinjing Gu, Zhiqiang Xu, Fan Lu, Liang Hu

**Affiliations:** ^1^Eye Hospital and School of Ophthalmology and Optometry, Wenzhou Medical University, Wenzhou, China; ^2^National Clinical Research Center for Ocular Diseases, Wenzhou, China

**Keywords:** dry eye, meibomian gland, corneal refractive surgery, OSDI questionnaire, NIBUT

## Abstract

**Purpose:**

To investigate the effect of the preoperative meibomian gland (MG) status on dry eye symptoms after corneal refractive surgery.

**Methods:**

This is a prospective, observational study. Subjects were enrolled and classified into 3 groups according to their MG loss grades. Ocular surface parameters were measured preoperatively and at 1, 3, and 6 months, postoperatively, including the ocular surface disease index questionnaire (OSDI), non-invasive tear film break up time (NIBUT), tear meniscus height and Schirmer I test. All the parameters were analyzed among the three groups, and different time points.

**Results:**

Seventy-eight patients were included in this study. The grade of MG loss varied from 0 to 2, thus the subjects were divided into group 1–3 corresponding to the MG loss. There were no significant differences in all parameters at baseline. The OSDI score increased in all groups at 1 month postoperatively and then decreased after other follow-ups. The OSDI was higher in group 3 than group 1 at all time points postoperatively (*P* = 0.005, 0.002, 0.034). Besides, it was higher in group 2 at 3 months and 6 months, compared with group 1 (*P* = 0.006, 0.029). The average NIBUT was shorter in group 3, compared with group 1 and group 2 since 1 month after surgery. At 1 and 3 month postoperatively, the grade of MG loss was positively correlated with the total OSDI and the vision-related scores. And it showed a positive correlation only with the environmental score at 6 months postoperatively.

**Conclusions:**

The dry eye discomfortable symptoms significantly differed post operatively according to their preoperative MG loss grade, though no difference was found at baseline. Dry eye was associated more with vision-related discomfort at first and environmental factors later.

## Introduction

Refractive surgery, as a procedure with high safety and stability, has been used globally in clinic for patients with certain amounts of ametropia (1). Although refractive surgery is common and produces excellent vision without the need for wearing glasses, some symptoms still remain and have huge implications for patient satisfaction and vision-related quality of life (2). Dry eye, as the most common complaint after refractive surgery, has been shown to interfere primarily with tear film dysfunction and influence patients' visual quality and satisfaction (3, 4). The study of dry eye and proper treatment after refractive surgery could also minimize the risk of regression (5).

Recent studies have shown that the incidence of dry eye after surgery is related to the basic status of the ocular surface before surgery. Albietz et al. demonstrated that the risk of chronic dry eye after laser *in situ* keratomileusis (LASIK) was associated with dry eye symptoms before surgery and the ocular surface management is important (5, 6). Furthermore, Chen et al. reported that the different thicknesses of the lipid layer before surgery are related to the signs of dry eye after surgery (7). Another study found the meibomian gland parameters were significantly worse in post-refractive surgery patients compared with normal controls (8). Meibomian gland dysfunction (MGD) has been wildly accepted as the major cause of dry eye (9, 10). However, when checking the subjects with meibomian gland dysfunction (MGD), there were only eight cases (11.7%) with MGD in the post-refractive group and seven MGD cases (13.3%) in the normal group (8). For many young patients who request corneal refractive surgery, MG status is largely overlooked when the sign is not severe enough to diagnose MGD (11). In recent years, studies have indicated that loss of the MG can result in instability of the tear film and tear film lipid layer (8, 11, 12) and Zhao et al. reported that even in asymptomatic children, MG deficiency is already present (13). In clinic, we found there are different degrees of MG loss among patients, but no notably different complaint of dry eye before refractive surgery. As dry eye is the main complaint after corneal refractive surgery, whether different MG status will aggravate dry eye in a different way after surgery is a clinical issue that urgently needs further research. To our knowledge, the effect of different preoperative MG loss on postoperative dry eye signs, and the potential relationship with the patient feelings has not been studied yet.

Therefore, we assessed the effect of preoperative MG status on dry eye symptoms and signs after corneal refractive surgery, which might provide clinical clues to guide clinical evaluations and preventions.

## Methods

The study adhered to the tenets of the Declaration of Helsinki and was approved by the Ethics Committee of Wenzhou Medical University. Informed consent was obtained from all patients prior to inclusion in the study. The exclusion criteria for the participants included eye inflammation, severe eye irritation, eyelid or ocular surface disease, contact lens wear for more than half a year, history of eye surgery, or systemic or eye disease that could interfere with tear film production or function. The data used in this study were obtained from the right eye of each subject. All patients in the study were enrolled from the refractive surgery center of the Eye Hospital of Wenzhou Medical University from April 2019 to December 2020.

The following examinations were performed before and at 1, 3, and 6 months after the operation: slit lamp biomicroscopy, ocular surface disease index questionnaire (OSDI), non-invasive tear film break-up time (NIBUT), tear meniscus height measurement (TMH), meibomian gland dropout score (MGDS), and the basic tear secretion Schirmer I test.

The OSDI questionnaire is used to measure a subject's subjective dry eye symptoms. It includes 12 questions that are rated 0 to 100 points, of which 0–12 is asymptomatic, 13–32 is mild and moderate, and 33–100 is severe symptoms. And the questions of OSDI could be divided into three parts as the ocular symptom score, the vision-related score and the environmental score. Each part represents a different sub-type of subjective dry eye symptoms. Both the total score and the sub-category score were recorded.

The NIBUT, TMH, and MGDS were measured using a Keratograph 5M (Oculus, Wetzlar, Germany). The TMH under the pupil was measured with a ruler that comes with the machine software. In addition, the first NIBUT (NIBUT-F) and average NIBUT (NIBUT-Ave) were measured.

The clear image of meibomian glands was captured by keratography 5M infrared, and we used Image J software to identify and calculate the absence of glands according to the image. The MGDS were evaluated by the ratio of the area of the meibomian gland loss to the total area using a 0–4 grading scale: grade 0 (no or minimal MG loss), grade 1 (<25% MG loss), grade 2 (25–50% MG loss), grade 3 (50–75% MG loss) and grade 4 (>75% MG loss). Subjects were classified into different groups according to their MG grade. Because the meibomian glands of the lower eyelid are irregular, we only obtained images of the upper eyelid meibomian glands of each right eye.

The Schirmer I test was performed by hooking a 35 mm x 5 mm Schirmer band on the edge of the lower eyelid and measuring the development of a length (mm) of wet paper in 5 min without anesthesia.

Statistical analysis was performed using SPSS23.0. Statistical differences among the groups were determined with the one-way ANOVA, and the LSD for determining differences between two groups was employed. Comparison of all time points among the same group was carried out with one-way repeated measures ANOVA and bonfferoni test. Pearson's correlation analysis was used to explore correlations between normal distribution variables and Spearman's rank correlation analysis was used to explore correlations between non-normal distribution variables.

## Results

Seventy-eight patients (78 eyes) were involved in the study. The MG loss of participants involved only differed from grade 0–2. Therefore, the patients were divided into three groups: Group 1: MG loss was grade 0; Group 2: MG loss was grade 1; Group 3: MG loss was grade 2. Specifically, 24 patients received femtosecond laser-assisted orthotopic keratomileusis (FS-LASIK, 8:8:8 in each group), 19 patients received small incision lenticule extraction (SMILE, 8:5:6 in each group), and 35 patients received Trans-Epithelial photorefractive keratectomy (Trans-PRK, 12:15:8 in each group). In our study, the operation type had no statistical effect on different groups. There was no statistical difference in the age, the gender and other basic parameters of subjects in each group (Table 1).

**Table 1:** Clinical parameters of patients before refractive surgery.

**Parameter**	**Group 1 (*n =* 28)**	**Group 2 (*n =* 28)**	**Group 3 (*n =* 22)**	***P*-value**
Age, years	23.39 ± 3.04	23.61 ± 4.86	25.00 ± 5.93	0.489
NIBUT-First, s	15.55 ± 3.67	14.72 ± 5.64	14.94 ± 4.46	0.796
NIBUT-Ave, s	18.15 ± 2.99	17.41 ± 4.75	16.23 ± 4.90	0.296
TMH, mm	0.36 ± 0.08	0.35 ± 0.08	0.38 ± 0.08	0.408
Schirmer's test I, mm	20.54 ± 8.55	19.14 ± 10.88	18.05 ± 9.87	0.668
OSDI score	6.62 ± 5.73	10.12 ± 10.54	9.09 ± 7.26	0.266
Gender	1.43 ± 0.50	1.29 ± 0.46	1.45 ± 0.51	0.408

*Data are presented as mean ± SD. NIBUT-First, first non-invasive tear breakup time; NIBUT-Ave, average non-invasive tear breakup time; TMH, tear meniscus height; OSDI, Ocular Surface Disease Index (OSDI) questionnaire*.

### OSDI Questionnaire

There were no significant difference in OSDI score at baseline among the three groups. At 1, 3, and 6 months after surgery, the OSDI scores of group 1 were 12.72 ± 1.34, 6.52 ± 0.85, 5.43 ± 0.70, respectively. The OSDI scores of group 2 were 15.18 ± 1.69, 11.31 ± 1.47, 8.11 ± 0.97 at each postoperative time points. And the OSDI scores of group 3 were 18.47 ± 1.45, 12.11 ± 1.60, 8.24 ± 1.16 at each postoperative time points.

**Figure 1:**
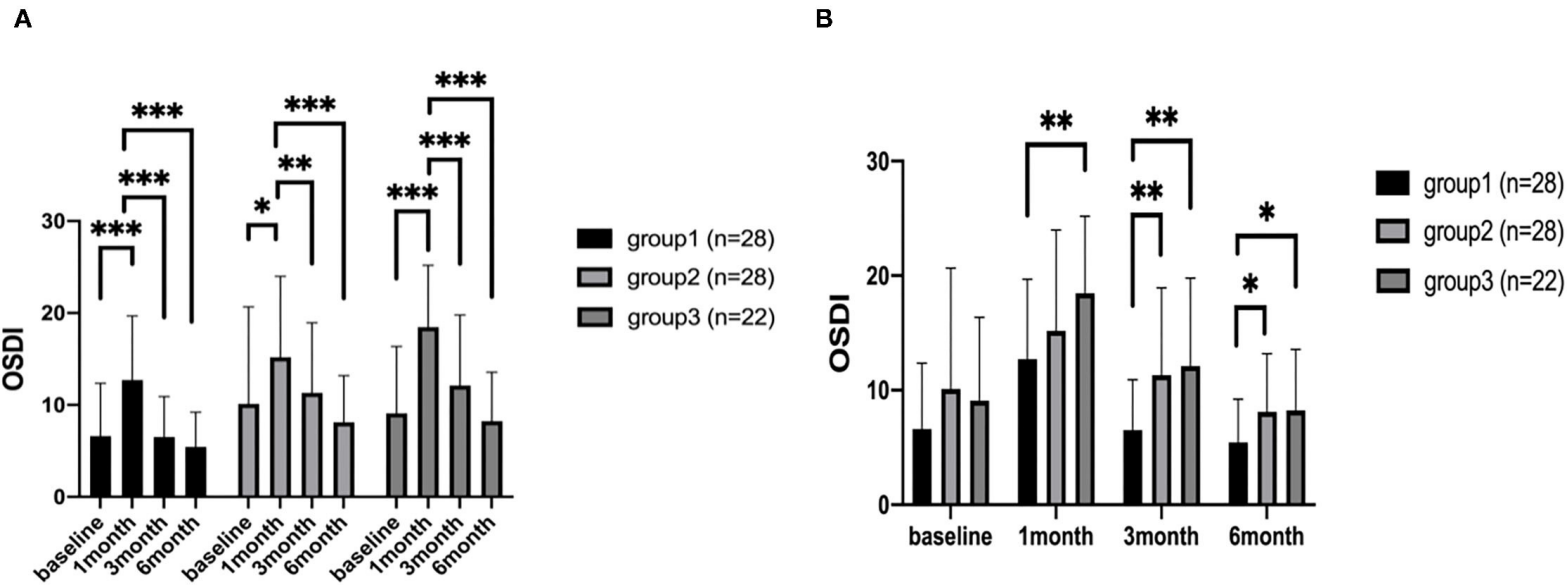
The OSDI scores of patients. The comparison of different time points measured preoperatively, at 1, 3, and 6 months after corneal refractive surgery **(A)**. The comparison among the three groups **(B)**. *P < 0.05, **P < 0.01, and ***P < 0.001 (OSDI, eye surface disease index questionnaire).

For comparisons between different time points, all three groups showed a significant increase at 1 month after corneal refractive surgery (*P* < 0.001, *P* = 0.012, *P* = 0.001, respectively), and the scores gradually decreased to preoperative levels at 3 and 6 months, postoperatively (Figure 1A). Significant differences were also found between 1 month after surgery and 3 month, 6 month postoperatively (Figure 1A). At 1 month postoperatively, all sub-category OSDI scores increased significantly in all three groups and gradually return to baseline levels at 3 months.

For comparison among the three groups, OSDI score in group 3 was higher than group 1 at all time points postoperatively (*P* = 0.005, 0.002, 0.034) (Figure 1B). However, it was higher only at 3 months and 6 months in group 2, compared with group 1 (*P* = 0.006, 0.029) (Figure 1B).

At 1 month postoperatively, the vision-related scores were significantly worse in group 3 compared with group 1 and group 2 (*P* = 0.007 and *P* = 0.037, respectively). And the vision-related scores of group 3 were significantly worse than group 1 at 3 months after surgery (*P* = 0.005). The environmental scores of group 3 were significantly higher than those of group 1 at 3 months and 6 months postoperatively.

### Ocular Surface Function Indexes

At 1, 3, and 6 months after surgery, the NIBUT-Ave of group 1 were 16.57 ± 0.69, 17.66 ± 0.72, 17.60 ± 0.58, respectively. The NIBUT-Ave of group 2 were 14.80 ± 0.84, 16.23 ± 0.86, 16.53 ± 0.89 at each postoperative time points. And the NIBUT-Ave of group 3 were 11.86 ± 0.79,13.57 ± 1.002, 12.82 ± 0.99 at each postoperative time points.

**Figure 2:**
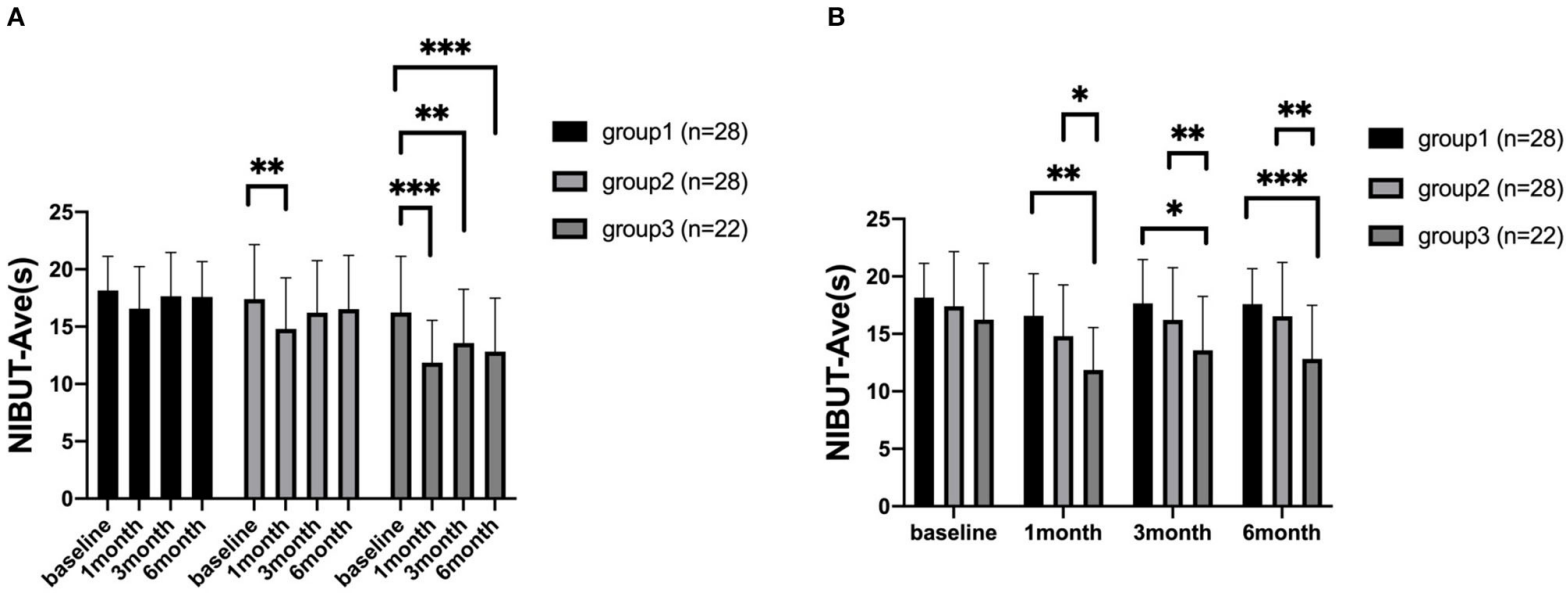
The average non-invasive tear breakup time (NIBUT-Ave) of patients. The comparison of different time points measured preoperatively, at 1, 3, and 6 months after corneal refractive surgery **(A)**. The comparison among the three groups **(B)**. *P <0.05, **P <0.01, and ***P <0.001 (NIBUT-Ave, The average non-invasive tear film break up time).

In group 1, the NIBUT-Ave showed no significant changes from the preoperative to different postoperative periods. In group 2, the NIBUT-Ave significantly decreased at 1 month (*P* = 0.001) and increased at 3 months and 6 months postoperatively. In group 3, the NIBUT-Ave significantly decreased at 1, 3, and 6 months postoperatively (*P* < 0.001, *P* = 0.002, *P* < 0.001, respectively), compared to the preoperative value (Figure 2A).

For comparison among the three groups, there were significant differences in NIBUT-Ave between group 1 and group 3 at 1, 3, and 6 months postoperatively (*P* < 0.001, *P* = 0.001, *P* < 0.001, respectively). The NIBUT-Ave was also shorter in group 3 than group 2, which showed a significant difference at each time points postoperatively (*P* = 0.017, *P* = 0.048, *P* = 0.008) (Figure 2B). For comparison of different time points, the NIBUT-F significantly worsened at 1 month postoperatively in all three groups, compared with the baseline values. For comparison among the three groups, there was only a significant difference between group 1 and group 3 at 6 month postoperatively. The measurements of TMH and Schirmer I test did not show significant postoperative differences (all *P* > 0.05).

### Correlation Analysis

Preoperatively, no correlation was found between MG loss and the OSDI scores or the ocular surface parameters. The grade of MG loss was positively correlated with the total OSDI and the vision-related scores at 1 months and 3 months postoperatively. The MG loss showed a positive correlation with the environmental score at 6 months postoperatively (Table 2).

**Table 2:** Correlation between the degree of meibomian gland loss and OSDI score at each time point before and after surgery.

		**OSDI**	**Ocular symptom score**	**Vision-related score**	**Environmental score**
Baseline	*R*-value	0.118	0.037	0.127	0.074
	*P*-value	0.326	0.761	0.294	0.542
1 month	*R*-value	0.258	0.015	0.319	0.090
	*P*-value	0.030^*^	0.904	0.007^**^	0.461
3 month	*R*-value	0.292	0.157	0.293	0.233
	*P*-value	0.014^*^	0.119	0.013^*^	0.050
6 month	*R*-value	0.250	0.152	0.098	0.263
	*P*-value	0.036	0.204	0.417	0.027^*^

*OSDI, Ocular Surface Disease Index (OSDI) questionnaire*;

* *p < 0.05*;

** *p < 0.01*.

## Discussion

Dry eye is one of the most common discomforts after corneal refractive surgery (14). It is characterized by a loss of homeostasis of the tear film. Loss of the MG may result in instability of the tear film (12, 15). We have already known that the ocular surface status is worsened after refractive surgery compared to normal controls (8). But for subjects with the insignificant symptoms and signs before surgery, whether MG loss would aggravate the dry eye status after corneal refractive surgery is still unknown. The level of MG loss associated with the worsening of the symptoms is not well-understood. Our results showed that the total OSDI and the ocular symptom scores increased significantly while the NIBUT-F and NIBUT- Ave decreased significantly at 1 month postoperatively. And the NIBUT-Ave was significantly shorter at all follow-ups postoperatively, which showed worsened instability of the tear film. It means that tear film instability was present with the patients' increasing discomfort after surgery. Previous studies have also proved that tear film break up time will be significantly reduced after refractive surgery (16, 17), which is consistent with our study. Furthermore, we found that the tear film instability of people with MG loss between 25 and 50% was worse than people without MG loss and people whose MG loss was within 25%. And for patients with higher preoperative MG loss grade, a significantly worse feeling of the total OSDI and the vision-related scores was present. So the preoperative MG loss influences the subjective feeling of dry eye discomfort, and tear film instability after surgery apparently, even though there is no significant difference at baseline.

As we didn't find any significant differences of the TMH and Schirmer's score among different groups, the post-refractive surgery dry eye differences may be correlated more with the tear film lipid layer rather than a decrease in aqueous tear production. Instability of the tear film caused by surgery was thought to be related to corneal nerve damage (18), or MG function deterioration (19). Refractive surgery may damage part of the corneal nerves which reducing corneal sensitivity and reflex blinking, lead to instability of tear film (12). It's also reported that ocular surface dryness symptoms may be related to corneal nerve damage (20). In our previous basic study, corneal sensitivity measured using the ocular surface esthesiometer showed the sensitivity decreased after surgery, thus less sensitive to dry eye discomfort (21). And in Han's study, deterioration of ocular surface function and meibomian gland function was found in patients after cataract surgery (19). They all suggested that the reduction of MG function caused by refractive surgery may contribute to chronic tear film dysfunction. And there were no structural changes during the 6 month follow-up in our study, which is consistent with previous study (8, 19, 22). So, we suppose that the effect of meibomian gland loss on the tear film can be superimposed with the effect of corneal refractive surgery, which aggravates the signs and symptoms of dry eye after surgery.

The MG loss grade showed no correlation with the OSDI scores and other ocular surface parameters before surgery in our study, which was also similar to several other cross-sectional dry eye studies (11, 23). However, some previous studies found significant correlations between MG loss and some tear film parameters, such as the lipid layer thickness (LLT), NIBUT, as well as subjective symptomatology (OSDI) in dry eye and MGD patients (24). Interestingly, the grade of MG loss in our study showed a significant positive correlation with the OSDI and vision-related scores after corneal refractive surgery at 1 month and 3 months, and it was significantly correlated with the environmental score at 6 months postoperatively. The results suggested the possibility that after some intervention, such as corneal refractive surgery, MG loss would influence the subjects' symptoms more apparently. The discomfort is related more to reading, driving at night, and working with a computer at an early period postoperatively, and it can be aggravated by environmental conditions, such as wind, low humidity or air conditioning use at the 6 month follow-up. All these revealed that after routine and successful corneal refractive surgery, the extent to which the patients experienced ocular discomfort differed according to their preoperative MG loss grade. Thus, more detailed information could be provided to patients after carefully evaluating the preoperative MG status and ocular surface status. For those with more MG loss, clinicians could explain that the vision-related discomfort may be more severe and several therapies could be tried, such as the application of hot compresses and good lid hygiene. At 3 months postoperatively and later, more attention could be paid to environmental factors.

In addition, studies have demonstrated that it is necessary to compare age-matched groups since there is great influence of aging on MG morphology and function, also a risk factor for dry eye after refractive surgery (25, 26). And age is studied to be the only factor associated with the LLT in normal subjects (27). In the present study, no differences were found in age among different MG loss groups. In this experiment, although we used three types of refractive surgery, the difference in surgical procedures had no statistical effect on different groups. For the post-operative treatment regimen, we use 0.1% fluorometholone eye drops, 4 times a day for the first 1 week, 3 times a day for the 2nd week, 2 times a day for the 3rd week, then once a day for the 4th week; routine use of artificial tears, 4 times a day, for at least 3 month, and use artificial tears as needed after for SMILE and LASIK. For T-PRK, the fluorometholone need to use longer and usually would be 4 times a day for the 1st month. Artificial tears is also routinely used.

Our present study also had limitations. We did not evaluate the ocular surface staining and meibography of inferior eyelid. Therefore, to understand more definite information about the relationship between MG loss and dry eye after refractive surgery, it is necessary to perform larger size with more examinations in future research.

To our knowledge, this is the first study to evaluate the preoperative MG status on dry eye discomforts of corneal refractive surgery patients. The study suggested that even the subjective symptom is similar in patients with various degree of MG loss before surgery, the feeling would turn to significantly different among the three groups after surgery. The better preoperative meibomian gland status, the better dry eye symptoms would be achieved after corneal refractive surgery. The vision-related discomfort would be the main complain at first and more attention could be paid to environmental factors. More research is needed to optimize the MG status during preoperative evaluation to deal with dry eye related problems and challenges, which would additionally improve the patient's quality of life and satisfaction.

## Data Availability Statement

The raw data supporting the conclusions of this article will be made available by the authors, without undue reservation.

## Ethics Statement

The study adhered to the tenets of the Declaration of Helsinki and was approved by the Ethics Committee of Wenzhou Medical University. The patients/participants provided their written informed consent to participate in this study.

## Author Contributions

LH: acquisition of data, drafting the article, final approval of the version to be published, and agreement to be accountable for all aspects of the work. FL: analysis and interpretation of data, drafting the article, final approval of the version to be published, and agreement to be accountable for all aspects of the work. QG: acquisition of data, revising it critically for important intellectual content, final approval of the version to be published, and agreement to be accountable for all aspects of the work. AL, HC, JG, and ZX: analysis and interpretation of data, revising it critically for important intellectual content, final approval of the version to be published, and agreement to be accountable for all aspects of the work. LC: substantial contributions to conception and design, revising it critically for important intellectual content, final approval of the version to be published, and agreement to be accountable for all aspects of the work. All authors contributed to the article and approved the submitted version.

## Funding

This research was supported by National Natural Science Foundation of China under Grant No. 82070933, Zhejiang Provincial Natural Science Foundation of China under Grant No. LY17H120005, and Wenzhou Basic Research Project, Y2020031.

## Conflict of Interest

The authors declare that the research was conducted in the absence of any commercial or financial relationships that could be construed as a potential conflict of interest.

## Publisher's Note

All claims expressed in this article are solely those of the authors and do not necessarily represent those of their affiliated organizations, or those of the publisher, the editors and the reviewers. Any product that may be evaluated in this article, or claim that may be made by its manufacturer, is not guaranteed or endorsed by the publisher.
